# ERK1/2-Egr-1 Signaling Pathway-Mediated Protective Effects of Electroacupuncture in a Mouse Model of Myocardial Ischemia-Reperfusion

**DOI:** 10.1155/2014/253075

**Published:** 2014-05-05

**Authors:** Juan Zhang, Jiangang Song, Jin Xu, Xuemei Chen, Peihao Yin, Xin Lv, Xiangrui Wang

**Affiliations:** ^1^Department of Anesthesiology, Renji Hospital, School of Medicine, Shanghai Jiao Tong University, 1630 Dongfang Road, Shanghai 200127, China; ^2^Department of General Surgery, Putuo Hospital, Shanghai University of Traditional Chinese Medicine, Shanghai 200062, China; ^3^Department of Anesthesiology, Shanghai Pneumology Hospital, School of Medicine, Tongji University, Shanghai 200433, China

## Abstract

Early growth response- (Egr-) 1 is an upstream master switch in controlling inflammatory responses following myocardial ischemia-reperfusion (I/R). Activation of extracellular signal-regulated protein kinase-1 and kinase-2 (ERK1/2) signaling is known to upregulate Egr-1. ERK1/2 pathway has been previously shown to mediate the therapeutic action of electroacupucture (EA). Thus, we hypothesized that EA would reduce myocardial I/R injury and inflammatory responses through inhibiting Egr-1 expression via the ERK1/2 pathway. Mice were pretreated with EA, U0126, or combination of EA and U0126 and then underwent 1 h myocardial ischemia and 3 h reperfusion. We investigated that EA significantly attenuated the I/R-induced upregulation of both Egr-1 and phosporylated-ERK1/2 (p-ERK1/2), decreased myocardial inflammatory cytokines including tumor necrosis factor-**α** (TNF-**α**) and interleukin-1**β** (IL-1**β**), and reduced the infarct size and the release of cardiac troponin I (cTnI). U0126 treatment also exhibited the same effect as EA on Egr-1 level and subsequent cardioprotective effects. There was no additive effect of cotreatment with EA and U0126 on the expression of Egr-1 and its downstream target genes (TNF-**α**, IL-1**β**) or serum cTnI level. Collectively, these observations suggested that EA attenuates myocardial I/R injury, possibly through inhibiting the ERK1/2-Egr-1 signaling pathway and reducing the release of proinflammatory cytokines.

## 1. Introduction

Acupuncture is a therapeutic technique that originated in China more than five thousand years ago [1]. Comparing with traditional manual acupuncture, electroacupuncture (EA) is more repeatable and adjustable. Accumulating evidences from experimental studies indicated that EA at selected acupoints [e.g., Neiguan (PC6)] can reduce myocardial ischemia-reperfusion (I/R) injury, as reflected by reducing release of myocardial enzyme such as cTnI and creatine phosphokinase (CPK) [2, 3], attenuating the frequency and severity of arrhythmias [4, 5] and decreasing infarct size [6–8]. More importantly, the beneficial effects of EA have also been observed in clinical settings, where it has resulted in reduced cTnI release, decreased C-reactive protein level, and shorter intensive care unit stay in both adult and pediatric patients receiving heart surgeries [2, 9]. Despite these visible benefits of EA, the underlying molecular mechanisms of EA-mediated cardiac protection remain unclear.

Early growth response- (Egr-) 1, a transcription factor, has been shown to be upregulated in the heart [10, 11] and initiate inflammation following I/R [12]. Using an Egr-1 antisense oligodeoxyribonucleotide or a catalytic deoxyribonucleic acid molecule (DNAzyme) to inhibit Egr-1 has been shown to reduce myocardial inflammation and protect heart function against the I/R [11, 13]. Studies using other disease models have also pointed to the regulation of Egr-1 by the ERK1/2 pathway. In lung I/R, Egr-1 requires the activation of ERK1/2 to exert its proinflammatory effects [14]. Upregulation of Egr-1 via the ERK1/2 pathway also contributes to the damage to pulmonary artery smooth muscle cells in a model of chronic hypoxia [15]. Recently, the ERK1/2 pathway has been implicated in the therapeutic effects of EA. It is reported that EA at PC6 acupoints alleviates cardiac hypertrophy after myocardial infarction by inhibiting the activation of ERK1/2 pathway [16]. Collectively, it suggests a link among EA, Egr-1, and ERK1/2 signaling pathway. Thus, we tested the hypothesis that EA attenuates myocardial I/R injury by inhibiting the ERK1/2-Egr-1 pathway.

## 2. Materials and Methods

### 2.1. Ethics Statements

All experimental protocols were approved by Animal Care Committee of Shanghai Jiao Tong University, Shanghai, China. All experiments conformed to the Guide for the Care and Use of Laboratory Animals published by the National Institutes of Health (NIH Publication, 8th Edition, 2011). For the experiments described here, we used a total of 153 male C57BL6 mice (8–10 weeks of age) from Sino-British SIPPR/BK Lab Animals (Shanghai, China). Animals were maintained under a 12/12 h light/dark cycle at 24°C  ±  1°C, with unrestricted access to standard food and water.

### 2.2. Experiment Design

The hypothesis schematic diagram and study protocol diagram are depicted in Figures S1 and S2 in Supplementary Material available online at http://dx.doi.org/10.1155/2014/253075. Three sets of experiments were conducted to investigate whether the ERK1/2-Egr-1 pathway is involved in the protective effects of EA against myocardial I/R injury. In experiment 1, thirty mice were used to examine the temporal profile of Egr-1 and p-ERK1/2 after 1 h ischemia followed by reperfusion of varying lengths of time (0, 3, 6, and 24 h; *n* = 6 for each time point). Based on the maximal expression of both Egr-1 and ERK1/2 observed in this experiment, reperfusion duration was set at 3 h for subsequent experiments (except for infarct size determination). Then we evaluated the effects of EA on Egr-1 and ERK1/2 expression during myocardial I/R, and mice were exposed to sham surgery (SHAM), I/R alone (IR), or EA prior to I/R (EA + IR, EA was performed 30 min prior to the surgery and lasted until the start of surgery) (*n* = 12/group). In experiment 2, the effects of U0126 [a highly selective inhibitor of ERK kinase; Cell Signaling Technology, Danvers, USA; 20 mg/kg, 1 h prior to the surgery, i.p. [17]] were compared to vehicle treatment (0.1% v/v DMSO) as well as I/R alone (*n* = 12/group). In experiment 3, mice received EA, U0126, or both treatments prior to the surgery (*n* = 9/group). Areas at risk (AAR) from the left ventricles (LV) were collected to measure the content of Egr-1, p-ERK1/2, and ERK1/2 using western blot, real-time PCR, and immunohistochemical staining. Myocardial levels of TNF-*α* and IL-1*β* were also measured. Serum was collected for the cTnI assay. Six mice per group were used to determine infarct size at 24 h after reperfusion.

### 2.3. Myocardial I/R Injury

Mice were anesthetized with 2% isoflurane (in 100% oxygen) under artificial ventilation using a rodent ventilator (Kent Scientific Co., Torrington, Connecticut, USA). The adequacy of anesthesia was verified using tail pinch. The heart was exposed at the fourth intercostal space, and the left anterior descending coronary artery (LAD) was occluded transiently using a 6–0 suture and reperfused for varying duration as described previously [18]. The incision was closed after the procedure, and the mice were allowed to recover from the anesthesia. Before the mice were sacrificed, AAR from the LV and blood from the inferior vena cava were collected for biochemical analyses. The body temperature was maintained at 37°C throughout the study.

### 2.4. Electroacupuncture

EA was delivered to PC6 acupoints bilaterally, at 1 mm above the wrist joint between the radius and ulna on the ventral surface of the forelimb [19]. Briefly, stainless needles were inserted to a depth of 3 mm and secured using plastic adhesive tape. Electrical stimulation (current of 1 mA, alternating dense and disperse mode, 2 Hz [0.6-ms pulse width] versus 100 Hz stimulation [0.2-ms pulse width], each lasting for 3 s) was delivered using an electrical stimulation device (HANS LH-202, Huawei Co., Beijing, China) for 30 min [20].

### 2.5. Determination of Infarct Size

Infarct size was evaluated by Evans blue and triphenyltetrazolium chloride (TTC) (Sigma-Aldrich Co., St. Louis, MO, USA) staining as described previously [21]. Briefly, LAD was religated at 24 h after the reperfusion followed by injection of 2% Evans blue into the aortic arch. The heart was sliced transversely into five blocks of equal thickness, incubated in 1% TTC for 15 min at 37°C, and fixed in 10% formalin overnight. Images were digitally captured using a microscope (DFC500, LEICA, Solms, Germany) and a digital camera (C-DSD230, Nikon, Tokyo, Japan). The LV area, AAR and infarct area (IA) were determined with planimetry software (Image J; National Institute of Health, Bethesda, USA) and adjusted for the weight. The ratio of AAR to LV area and IA to AAR was calculated.

### 2.6. Western Blot Analysis

The LV was frozen in liquid nitrogen and stored at −80°C until homogenization on ice using an EDTA-free buffer containing protease inhibitor cocktail (Roche Ltd., Basel, Switzerland). The homogenate was centrifuged for 15 min at 12,000 rpm at 4°C. Protein concentration of the supernatant was determined using a BCA kit (Thermo Scientific, Middletown, USA). Equal amounts of protein (40 *μ*g) were fractionated in 12% SDS-polyacrylamide gels and transferred onto nitrocellulose membranes. The membranes were blocked with 5% nonfat dry milk and 0.01% Tween-20 in Tris-buffered saline (TBS) at pH 7.6 prior to incubation with monoclonal antibodies against Egr-1, p-ERK1/2, ERK1/2 (Cell Signaling Technology, Danvers, USA), or GAPDH (Kangcheng, Shanghai, China) at 4°C overnight. After incubation with an anti-rabbit secondary antibody (Biotime, Shanghai, China), the bands were visualized and analyzed using a BIO-RAD system (Molecular Imager ChemiDoc XPS+, Hercules, USA). GAPDH was used as an internal control.

### 2.7. Quantitative Real-Time Polymerase Chain Reaction

Egr-1 mRNA in the LV was measured using quantitative real-time polymerase chain reaction (qRT-PCR) with SYBER Premix Ex Taq and Primescript RT reagent Kit (Takara, Otsu, Japan) and expressed as 2^−ΔΔCt^ (relative fold change). GAPDH was used as an internal control. The primers for Egr-1 were forward 5′-GCCTTAAGGGGGTAGGAGTG-3′ and reverse 5′-CCTCTTCCTCATCGTGCTCT-3′. The primers for GAPDH were forward 5′-GGTTGTCTCCTGCGACTTC-3′ and reverse 5′-CCTGTTGCTGTAGCCGTATTCAT-3′. The PCR was conducted using a standard cycle: 95°C for 10 minutes, followed by 40 cycles of 95°C for 15 seconds, 56°C for 30 seconds, and 72°C for 30 seconds.

### 2.8. Immunohistochemical Staining

The LV was fixed in 4% paraformaldehyde and embedded in paraffin for sectioning into 4 *μ*m sections. After antigen retrieval, the sections were incubated with a primary antibody against Egr-1 followed by incubation with a biotin-conjugated secondary antibody and color reaction with avidin-peroxidase (ABC kit) and a DAB substrate kit (Vector Laboratories, Burlingame, USA). The sections were counterstained with hematoxylin. Images were digitized using a microscope (BX-51; Olympus, Tokyo, Japan) and analyzed using Image J software. The measurement was performed by two researchers blinded to the treatment condition.

### 2.9. ELISA

TNF-*α* and IL-1*β* in myocardial homogenate were examined using ELISA reagent kits (R&D systems, Minneapolis, USA). Serum cTnI was determined using an ELISA kit (Life Diagnostics, West Chester, USA).

### 2.10. Statistical Analysis

Data are expressed as the mean ± SEM and statistically analyzed by the Statistical Package for the Social Sciences (SPSS, Version 16.0; SPSS Inc., Chicago, USA). Independent Student's *t*-test was used when comparing the infarct size between IR and EA + IR group. All the other data were analyzed with one-way ANOVA followed by Turkey's post-test. *P* < 0.05 was considered statistically significant.

## 3. Results

### 3.1. EA Pretreatment Inhibited Myocardial Egr-1 and p-ERK1/2 Expression, Decreased Inflammatory Cytokines, and Reduced Infarct Size

**Figure 1:**
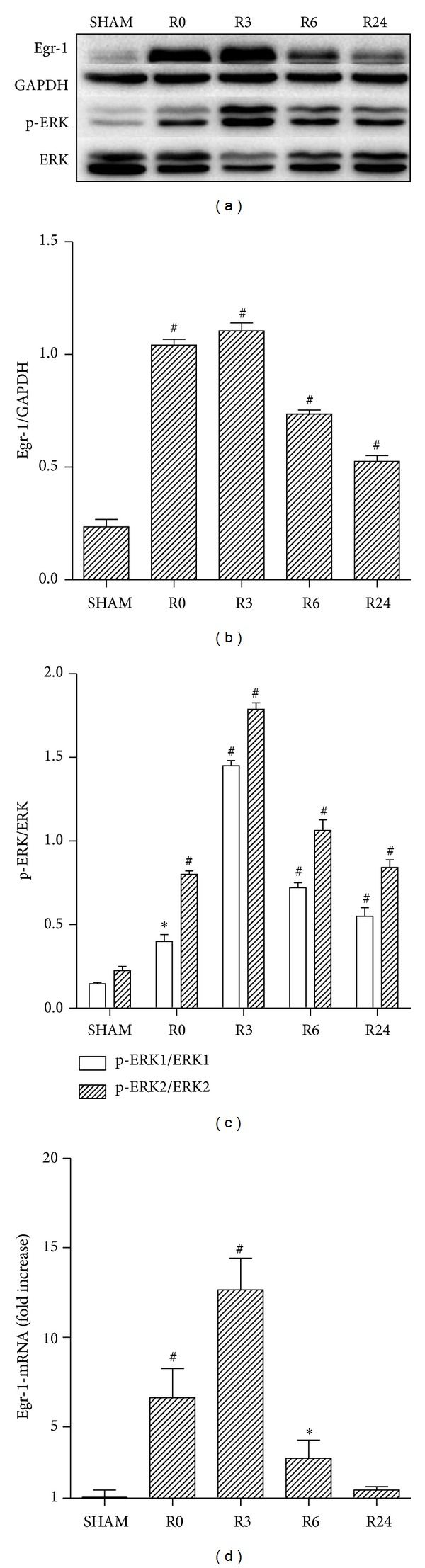
Comparison of the myocardial Egr-1and p-ERK1/2 expression at different time points after I/R. The protein levels of Egr-1and p-ERK1/2 at varying reperfusion time points (R0, R3, R6, and R24) were determined by western blot (a) and the corresponding densitometric analysis is shown in (b, c). Besides, the mRNA levels of Egr-1 were measured by qRT-PCR with data presented as relative fold increase versus sham control (d). For Egr-1 and p-ERK1/2, nearly all time points exhibit significant increase comparing with sham controls and peak levels are observed at R3 time point (*P* < 0.001, R3 versus SHAM). Six mice were sacrificed at each time points for protein and mRNA determination. R0 = ischemia for 1 h; R3 = ischemia for 1 h + reperfusion for 3 h; R6 = ischemia for 1 h + reperfusion for 6 h; R24 = ischemia for 1 h + reperfusion for 24 h. Data are presented as mean ± SEM; *P < 0.05 and ^#^ *P* < 0.01 versus sham control.

**Figure 2:**
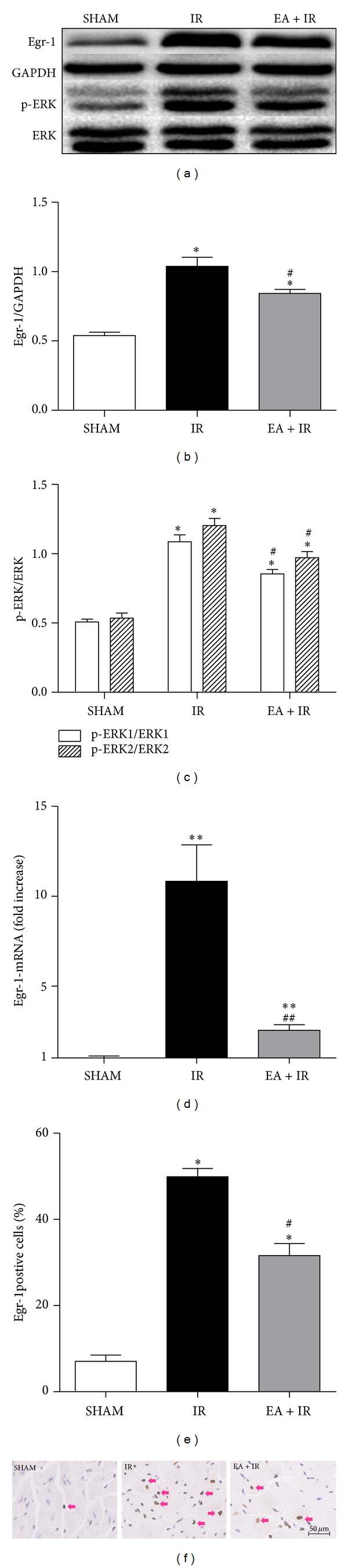
EA inhibited Egr-1 expression and ERK1/2 activation in myocardium undergoing myocardial I/R. Mice were divided into 3 groups: SHAM, IR (myocardial I/R), and EA + IR (EA stimulation was performed 30 min before myocardial I/R surgery and lasted for 30 min). After 3 h of reperfusion, the animals were sacrificed and the protein levels of Egr-1and p-ERK1/2 were measured by western blot (a) and densitometric analysis is shown in panel (b-c) (*n* = 3/group). The mRNA levels of Egr-1 in these three groups are represented as the relative fold increase versus sham controls (d, *n* = 3/group). Immunohistochemical staining of Egr-1 was performed and the quantitation results of Egr-1positive cells were shown in panel (e). Representative images were shown in panel (f). Pink arrows indicate Egr-1positive cells. Scale bar = 50 *μ*m. *P < 0.05 versus sham control; ^#^ *P* < 0.05 versus IR; **P < 0.01 versus sham control; ^##^ *P* < 0.01 versus IR.

**Figure 3:**
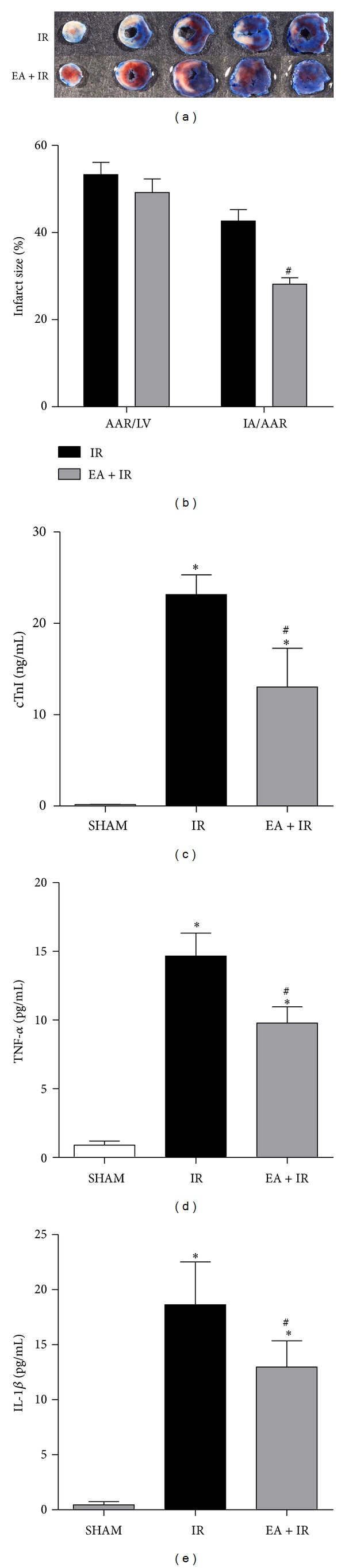
EA attenuated I/R-induced myocardial tissue damage and inflammatory response. After 24 h of reperfusion, Evans blue/TTC staining was applied to measure the infarct size ((a-b) *n* = 6/group). AAR/LV: area at risk/left ventricle area; IA/AAR: infarct area/area at risk. After 3 h of reperfusion, the serum cTnI level (c, *n* = 6/group) and the myocardial levels of TNF-*α* and IL-1*β* ((d-e), *n* = 3/group) were determined using ELISA. *P < 0.01 versus sham control; ^#^ *P* < 0.05 versus IR alone.

Varying reperfusion time points demonstrated changes in mRNA and protein levels of Egr-1 paralleled that of p-ERK1/2. Nearly all time points exhibiting significant increases from Sham controls and peak levels were observed after 3 h of reperfusion (*P* < 0.001 versus SHAM; Figures 1(a)–1(d)). Using 3 h of reperfusion, we tested the effects of EA on myocardial Egr-1 and p-ERK1/2 levels. EA treatment significantly attenuated the I/R-induced increase in Egr-1 (*P* = 0.036 for protein, *P* < 0.001 for mRNA; EA + IR versus IR) and p-ERK1/2 (*P* = 0.030 for p-ERK1, *P* = 0.037 for p-ERK2; EA + IR versus IR; Figures 2(a)–2(d)). Immunohistochemical staining also revealed that EA attenuated Egr-1 expression in response to I/R (Figures 2(e)-2(f)). In addition, the infarct size (IA/AAR) is significantly smaller in EA pretreatment group than the I/R alone group (28.15 ± 1.59% versus 42.64 ± 2.83%, *P* = 0.019; Figures 3(a)-3(b)). EA decreased the cTnI release in the serum (23.16 ± 1.25 versus 13.03 ± 1.89 ng/mL for I/R controls, *P* = 0.006; Figure 3(c)) and led to a reduction in myocardial TNF-*α* (14.66 ± 1.67 versus 9.78 ± 1.19 pg/mL for I/R controls, *P* = 0.043, Figure 3(d)) and IL-1*β* levels (18.62 ± 1.95 versus 12.97 ± 1.18 pg/mL for I/R controls, *P* = 0.035, Figure 3(e)). These findings indicated a protective effect of EA against the myocardial damage and inflammatory injury.

### 3.2. Activation of the ERK1/2 Pathway Is Responsible for Egr-1 Upregulation and Myocardial Injury following I/R 

**Figure 4:**
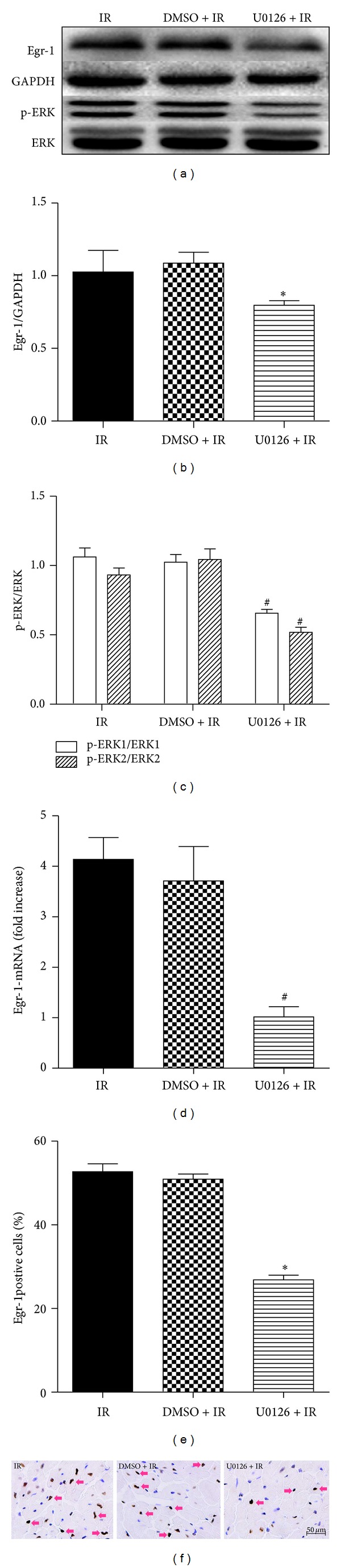
ERK1/2 activation is responsible for Egr-1expression during myocardial I/R injury. Mice received U0126 (an inhibitor of ERK1/2 kinase, 20 mg/kg, i.p.) or its vehicle 0.1% v/v DMSO treatment before surgery. As described previously, myocardial expression of Egr-1 and p-ERK1/2 was measured using western blot (a). The corresponding densitometric analysis is shown in (b-c) (*n* = 3/group). The mRNA levels of Egr-1 are shown as fold increase versus U0126 + IR (d, *n* = 3/group). Immunohistochemical staining of Egr-1 was performed and the quantitation results of Egr-1positive cells were shown in panel (e). Representative images were shown in panel (f) (3 mice /group). Pink arrows indicate Egr-1positive cells. Scale bar = 50 *μ*m. *P < 0.05 and ^#^ *P* < 0.01 versus DMSO + IR control.

**Figure 5:**
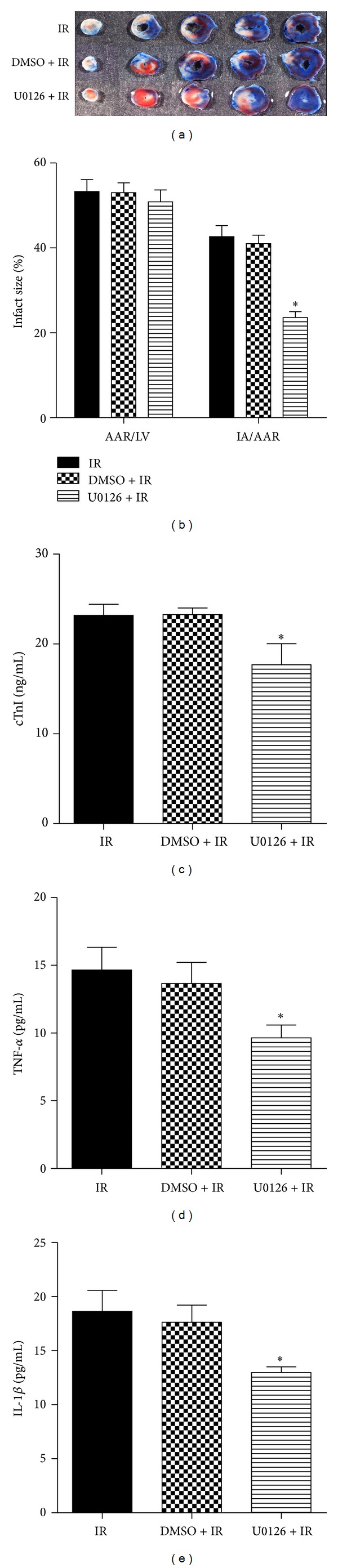
Inhibiting ERK1/2 activation with U0126 protected the myocardium against I/R injury. After 24 h of reperfusion, Evans blue/TTC staining was applied to measure the infarct size ((a-b) *n* = 6/group). AAR/LV: area at risk/left ventricle area; IA/AAR: infarct area/area at risk. After 3 h of reperfusion, the serum cTnI level (c, *n* = 6/group) and the myocardial levels of TNF-*α* and IL-1*β* ((d-e) *n* = 3/group) were determined using ELISA. *P < 0.05 versus DMSO + IR.

In order to determine the effects of ERK1/2 inhibition on I/R injury, we used U0126 to block the activation of ERK1/2 in animals undergoing I/R surgery. U0126 treatment significantly inhibited p-ERK1/2 (*P* = 0.003, U0126 + IR versus DMSO + IR; Figures 4(a) and 4(c)). Additionally, U0126 treatment significantly decreased both Egr-1 protein (*P* = 0.026) and mRNA levels (*P* < 0.001, U0126 + IR versus DMSO + IR; Figures 4(a), 4(b), and 4(d)). Immunohistochemical analysis revealed fewer myocardial Egr-1-positive cells in mice receiving U0126 in comparison to the vehicle control (Figures 4(e)-4(f)). In addition, the infarct size (IA/AAR) in the U0126 + IR group was significantly reduced when compared with the DMSO + IR group (*P* = 0.029, 23.62 ± 1.43% versus 41.03 ± 2.03%; Figures 5(a)-5(b)) as well as the serum cTnI levels (*P* = 0.034, 23.24 ± 0.74 versus 17.67 ± 2.33 ng/mL; Figure 5(c)). The myocardial TNF-*α* and IL-1*β* levels in U0126 + IR group were attenuated when compared with DMSO + IR group (*P* = 0.035 for TNF-*α*: 13.66 ± 1.56 versus 9.65 ± 0.94 pg/mL; *P* = 0.043for IL-1*β*: 17.62 ± 1.59 versus 12.97 ± 0.54 pg/mL; Figures 5(d)-5(e)).

### 3.3. Combination of EA and ERK1/2 Inhibition Did Not Further Enhance Cardiac Protection against I/R Injury

**Figure 6:**
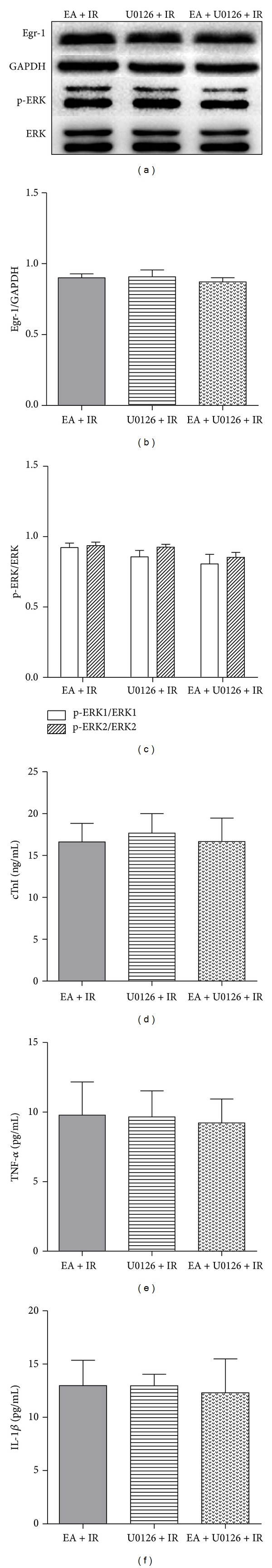
Combination of EA with ERK1/2 inhibitor did not produce more protection against myocardial I/R injury. Combination of EA and U0126 treatment was conducted to investigate the additive effects on ERK1/2/Egr-1 downregulation or the cardiac protective role. Western blot bands and corresponding densitometric analysis of Egr-1 and p-ERK1/2 are shown in (a–c) (*n* = 3/group). The myocardial levels of TNF-*α* and IL-1*β* ((d-e), *n* = 3/group) as well as the serum level of cTnI (f, *n* = 6/group) were determined.

As shown previously, both EA and U0126 treatment had favorable effects on myocardial damage and inflammation processes when given separately. However, effects of combined treatment of EA and U0126 did not differ from either EA or U0126 alone when comparing myocardial Egr-1 and p-ERK1/2 expression (*P* > 0.05; Figures 6(a)–6(c)). Additionally, EA + U0126 did not further affect the myocardial level of TNF-*α*, IL-1*β*, or serum cTnI levels in comparison to EA or U0126 alone (*P* > 0.05; Figures 6(d)–6(f)).

## 4. Discussion

The current study confirmed the cardiac protection of EA against I/R reflected by reduced infarct size and lower serum cTnI level, which is consistent with work done by our group and others [3, 22, 23]. Moreover, we demonstrated that EA at PC6 acupoints significantly attenuated I/R-induced upregulation of Egr-1 as well as ERK1/2 activation in the myocardium. Additionally, blocking ERK1/2 activation with U0126 elicited a significant decrease in Egr-1 expression and myocardial I/R injury. These data indicated that the cardiac protective effect of EA on myocardial I/R may be partially mediated by the attenuation of ERK1/2 activation and decrease in the downstream Egr-1expression in the myocardium.

Egr-1 is initially linked to the control of cell growth, survival, and transformation [24]. Recently, it has been implicated as a “master switch” in the injury response in a variety of models including vascular restenosis as well as I/R injury of many organs such as lung, heart, gut, and kidney [14, 25–28]. However, the upstream components capable of activating Egr-1 in myocardial I/R is still unclear. In a mouse lung I/R model, the ERK1/2 pathway was demonstrated to trigger Egr-1 expression and subsequent inflammatory damage [14]. In the current study, the change in Egr-1 after I/R parallels that of p-ERK1/2 at both mRNA and protein levels. After blocking the activation of ERK1/2 with U0126, the Egr-1 expression was significantly reduced and the myocardial injury was attenuated as well. Thus, our findings suggest that ERK1/2 is an important upstream signal for modulating Egr-1 expression underlying myocardial ischemia stress.

Although ERK1/2 has been generally reported to be an important member of prosurvival kinases in ischemic preconditioning [29], its role in myocardial ischemia stress remains controversial. In both I/R-injured rat hearts and hypoxia-reoxygenation-injured cardiomyocytes, inhibiting ERK1/2 activation reverses the reactive oxygen species production and intracellular Ca^2+^ overload [30]. Furthermore, treating hypertrophic H9c2 cells with U0126 reduces the DNA fragmentation and nuclear condensation [31]. These findings are consistent with our data that blocking the activation of ERK1/2 with U0126 alleviates myocardial I/R injury.

EA can regulate the activation of ERK1/2 to produce anti-inflammatory [32] and analgesic effects [33, 34]. In a middle cerebral artery occlusion (MCAO) model, EA at either GV20 (Baihui) or DU26 (Renzhong) acupoints increases the phosphorylation of ERK1/2 in the ischemic cortex and hippocampus [35]. In contrast, in animal models of both chronic constriction injury (CCI) and cardiac hypertrophy [16, 33], EA reduced the expression of p-ERK1/2. Consistently, in the current study, we found the I/R-induced myocardial expression of both p-ERK1/2 and Egr-1 was downregulated by EA at PC6 acupoints. Then we combined EA with the ERK1/2 inhibitor U0126 to investigate if they have an additive effect. However, the combined protocol did not further decrease p-ERK1/2 or Egr-1 expression or protect the myocardium. We speculate that ERK1/2-Egr-1 pathway might be a common target for both EA and U0126, and either EA or U0126 may have already exerted their maximal effects in our present experiment conditions.

In summary, EA treatment at PC6 (Neiguan) acupoints decreased infarct size, reduced the release of proinflammatory cytokines, and inhibited ERK1/2 activation and Egr-1expression in a mouse myocardial I/R injury model. The inhibition of ERK1/2-Egr-1signaling pathway may be, at least in part, responsible for the cardioprotective effects of EA against I/R injury.

## Supplementary Material

We hypothesized that EA would reduce myocardial I/R injury and inflammatory responses through inhibiting Egr-1 expression via the ERK1/2 pathway (see the schematic diagram in Fig. S1). In order to demonstrate this hypothesis, we designed the experiments as follow (see Fig. S2)
