# Supplementary material for: ERK1/2-Egr-1 Signaling Pathway-Mediated Protective Effects of Electroacupuncture in a Mouse Model of Myocardial Ischemia-Reperfusion
Source: Evid Based Complement Alternat Med. 2014 May 5;2014:253075. doi: 10.1155/2014/253075 (PMC4026842; doi:10.1155/2014/253075)
Supplement: Supplementary file 1 — We hypothesized that EA would reduce myocardial I/R injury and inflammatory responses through inhibiting Egr-1 expression via the ERK1/2 pathway (see the schematic diagram in Fig. S1). In order to demonstrate this hypothesis, we designed the experiments as follow (see Fig. S2) [file 253075.f1.pdf]

## Supplementary Figures

## Fig.S1

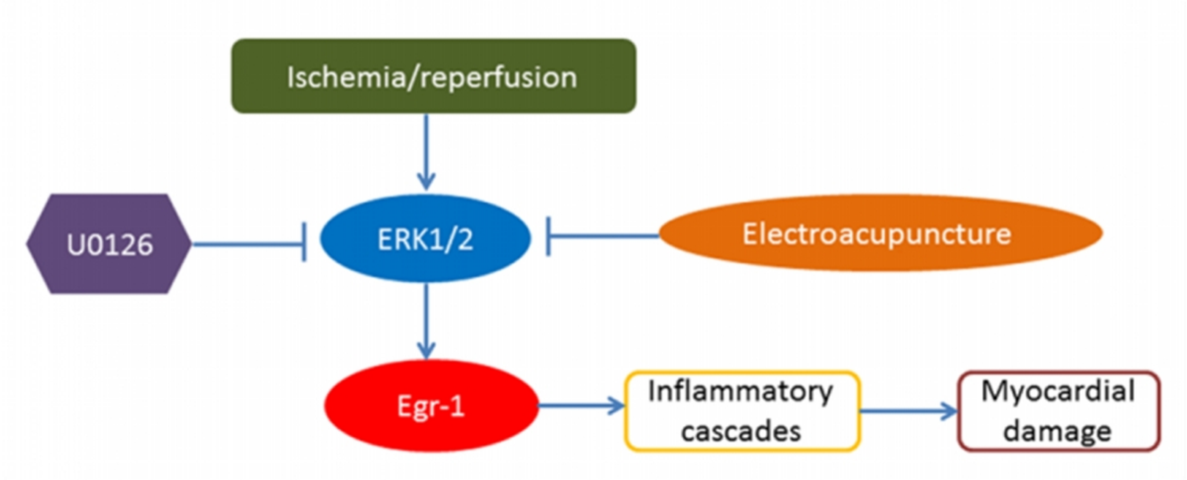


**Fig. S1: The hypothesis schematic diagram**

ERK1/2= Extracellular signal-regulated kinase 1/2; U0126= an ERK1/2 kinase inhibitor; Egr-1= Early growth response-1

## Fig.S2

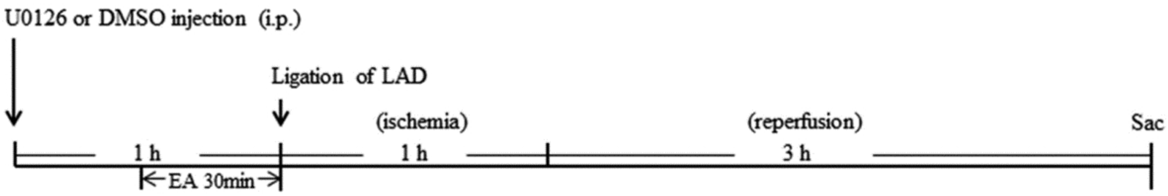


**Fig. S2: The study protocol**

Groups in experiment 1: SHAM (sham surgery), IR (myocardial I/R) and EA+IR (EA + myocardial I/R); Groups in experiment 2: IR (myocardial I/R), U0126+IR (U0126 injection + myocardial I/R), DMSO+IR (DMSO injection+ myocardial I/R); Groups in experiment 3: EA+IR (EA + myocardial I/R), U0126+IR (U0126 injection + myocardial I/R), EA+U0126+IR (EA + U0126 injection + myocardial I/R). U0126= an ERK1/2 kinase inhibitor; DMSO= dimethylsulfoxide (vehicle treatment for U0126); EA= electroacupuncture; LAD= left anterior descending coronary artery; IR=ischemia-reperfusion.
